# Severe emphysematous pyelonephritis as a complication of klebsiella pneumonia and uncontrolled diabetes mellitus

**DOI:** 10.1259/bjrcr.20220158

**Published:** 2023-10-18

**Authors:** Mihir Rao, Jina Pakpoor, Douglas Pendse, Conrad von Stempel

**Affiliations:** 1 Harrogate and District NHS Foundation Trust, Harrogate, United Kingdom; 2 University College London Hospitals NHS Foundation Trust, London, United Kingdom

## Abstract

A female in her 40s, with poorly controlled Type I diabetes mellitus, was brought to our tertiary hospital by ambulance after being found drowsy. Six days prior, she had self-discharged from the Intensive Care Unit whilst being treated for a Klebsiella pneumonia. At re-admission, she had an acute kidney injury with abdominal pain and clinical features of sepsis. Her presentation was attributed to ongoing *Klebsiella pneumoniae* infection; however, a chest radiograph showed marked improvement of pulmonary consolidations and an unusual subdiaphragmatic gas pattern. A CT scan demonstrated severe bilateral emphysematous pyelonephritis. The patient was unfit for bilateral nephrectomy and was medically managed in the Intensive Care Unit for 41 days, before transfer to a specialist renal unit for life-long haemodialysis. This case highlights the importance of considering emphysematous pyelonephritis in patients presenting with uncontrolled diabetes mellitus and acute kidney injury and/or infection, the role of imaging in its diagnosis, and the challenges of complex social circumstances in health management.

## Background

Emphysematous pyelonephritis is a rare and serious complication of uncontrolled diabetes mellitus, characterized by intra- or perinephric gas and with a high mortality if not treated early on.^1^ Due to low prevalence and the need for diagnostic imaging, a high index of suspicion is required, in particular when there are concurrent signs of sepsis or acute kidney injury (AKI) with uncontrolled diabetes mellitus. High urine and tissue glucose levels in diabetes mellitus promote bacterial growth in the urinary tract, and therefore the most common aetiology of emphysematous pyelonephritis is as a complication of a urinary tract infection.^1^ While *Klebsiella pneumoniae*, a gas-forming organism, is one of the most common organisms causing a urinary tract infection, we present a case of emphysematous pyelonephritis in a patient with a pulmonary *Klebsiella pneumoniae* infection. This case highlights the importance of a structured approach to image interpretation and the significance of holistically managing critically unwell patients, whilst also adding to the limited literature surrounding emphysematous pyelonephritis by reviewing the commonly associated clinical presentations and radiological findings.

## Clinical presentation

A female in her 40s was brought into Accident & Emergency (A&E) by ambulance after being found to be drowsy at home by a family member. The paramedics noted the patient to be hypoxic with an SpO2 of 85% and measured a blood glucose level of 6.8 mmol l^−1^.

As the patient was drowsy, a thorough history could not be taken at the time. However, from the patient’s medical records, it was found that she had recently self-discharged from the Intensive Care Unit (ICU) 6 days prior whilst being treated for a Klebsiella pneumonia. At the time of self-discharge, the patient had hyperglycaemic metabolic acidosis with ketones in the normal range. She had an extensive past medical history of non-compliance to medication and medical advice, resulting in poorly controlled Type I diabetes mellitus (on insulin) and malnourishment, with prior complications including neuropathic arthropathy, urinary retention, chronic pancreatitis, low body mass index, a pressure ulcer, and multiple fragility fractures. A previous history of pulmonary aspergillosis was also noted.

On hospital assessment, the patient had significantly reduced consciousness but a patent airway. She had a respiratory rate of 26 breaths per minute and SpO2 of 96% on 4 L of oxygen. On auscultation, crackles were heard in the left lung field. The patient was tachycardic (heart rate of 126 beats per minute), slightly hypotensive (blood pressure of 108/50 mmHg) and pyrexial (temperature of 37.9°C), but was well perfused. She was visibly cachectic and had dry mucous membranes.

## Investigations

**Figure 1.**
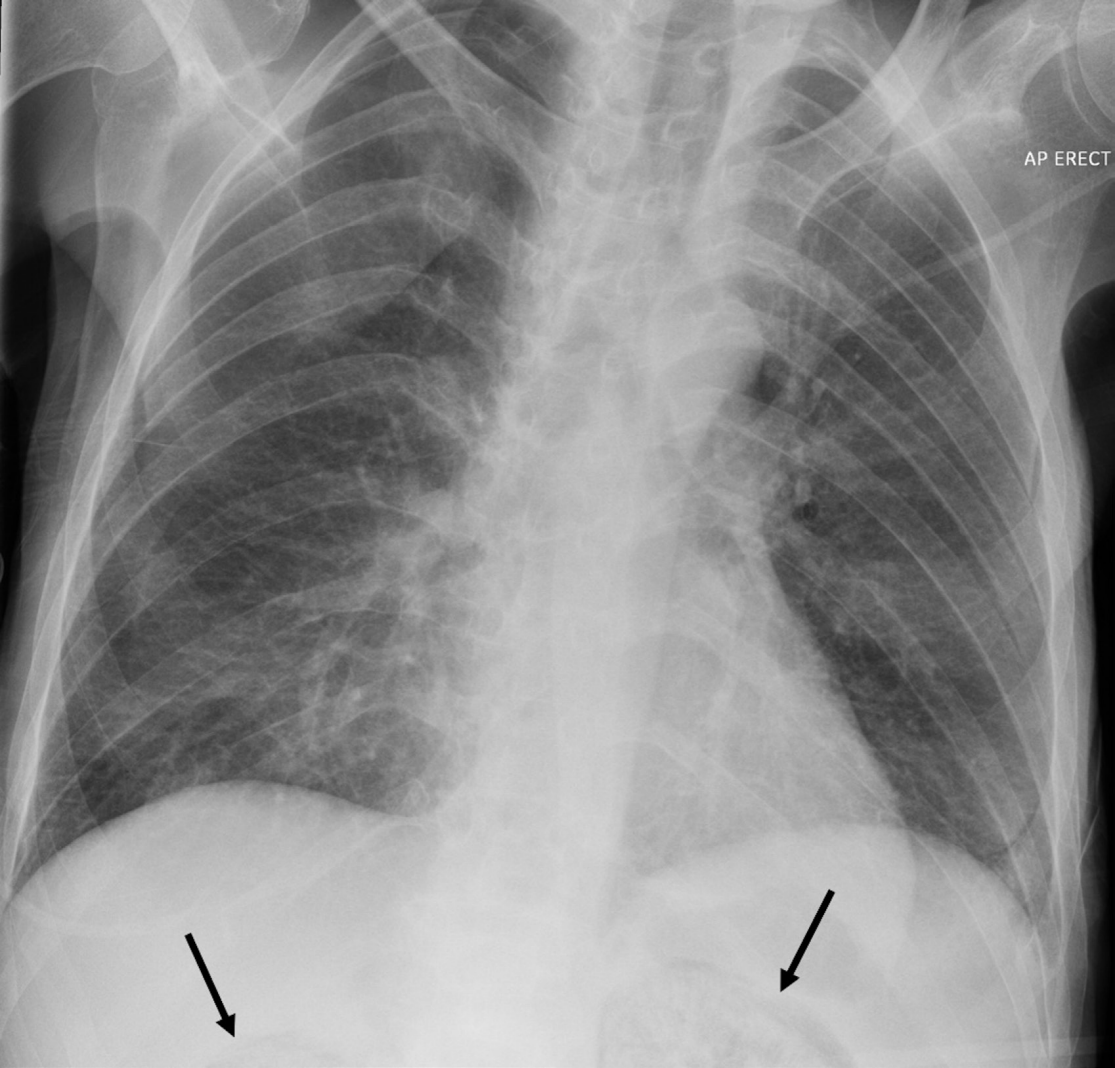
Portable erect chest radiograph demonstrating the presence of gas in the partially imaged upper pole of the kidneys.

Whilst in A&E Resus, the patient underwent a septic screen that included a mobile erect chest radiograph (**Figure 1**). This demonstrated diffuse hazy shadowing, attributed to atelectasis and incomplete resolution of the patient’s previous pneumonia.

Blood tests as part of the septic screen demonstrated a raised white cell count (17.6 × 10^9^  l^−1^) with neutrophilia (16.1 × 10^9^  l^−1^) and relative lymphopenia (0.8 × 10^9^  l^−1^) and raised c-reactive protein (400 mg l^−1^), suggestive of a bacterial infection. A blood culture and a urine culture showed the presence of Gram-negative rods of *Klebsiella pneumoniae*. A coagulation screen demonstrated raised prothrombin time (20.4 s), raised activated partial thromboplastin time (52 s) and raised INR (1.8), consistent with disseminated intravascular coagulation (DIC). The patient was also found to be in severe AKI, with severe oliguria, a creatinine of 363 µmol l^−1^ and GFR of 12 ml/min/1.73 m^2^. Six days previously, her creatinine was 35 µmol l^−1^, and GFR >90 ml/min/1.73 m^2^.

Considering the patient’s unexplained clinical features of sepsis and complaints of abdominal pain, a CT scan of the abdomen and pelvis was requested to look for an intra-abdominal infective source.

**Figure 2.**
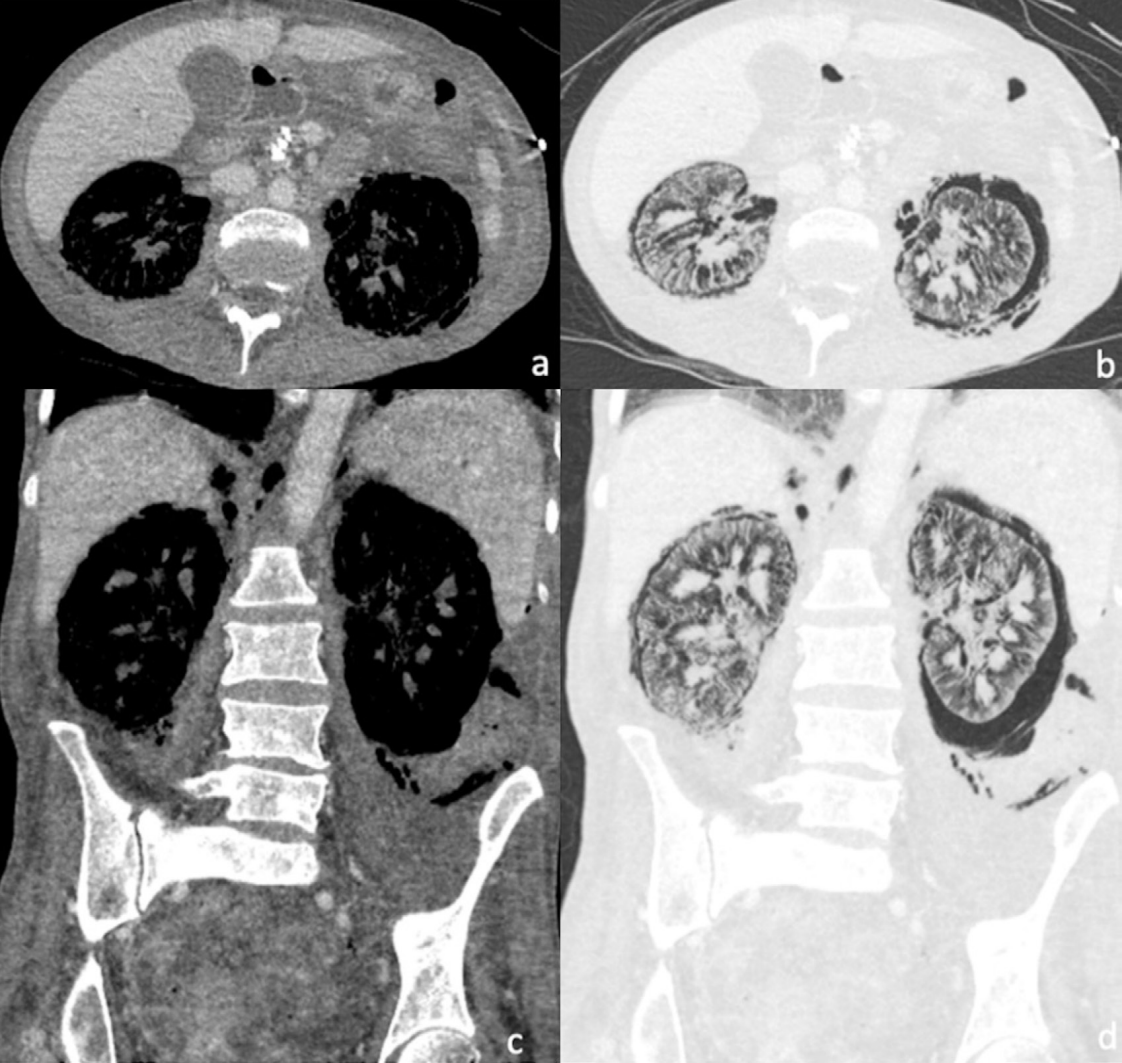
(a–d) I.V. contrast-enhanced CT abdomen and pelvis demonstrating extensive bilateral gaseous replacement of the normal renal parenchyma and perinephric space, consistent with emphysematous pyelonephritis. Free gas can be seen to track within the retroperitoneum, extending superiorly into the mediastinum and inferiorly along the left paracolic gutter and into the pelvis. (a) and (c) are set on soft tissue windows. (b) and (d) are on lung windows.

Axial and coronal CT views of the abdomen and pelvis (Figure 2a–d) demonstrated markedly abnormal, gas filled kidneys bilaterally, consistent with severe emphysematous pyelonephritis; there was almost no discernible normal renal tissue, concerning for complete renal failure. Gas extended to the non-dilated ureters bilaterally, and within the collapsed bladder lumen, but not within the bladder wall. Additionally, gas extended to a gas-filled collection in the right psoas major and through Gerota’s fascia and left paracolic gutter into the left pelvis.

## Differential diagnosis

Given the patient’s history of uncontrolled diabetes mellitus, microbiology findings and radiological CT findings, a diagnosis of severe emphysematous pyelonephritis was made. 95% of patients with emphysematous pyelonephritis have underlying diabetes mellitus, although other causes of immunocompromise are sometimes associated.^2,3^ *E. coli*, *Klebsiella* and *Proteus* species are the most commonly isolated organisms in this condition.^3^ The patient’s recent Klebsiella pneumonia, *Klebsiella* positive blood culture and non-compliance with medical advice and treatment, put her at a higher risk of developing further Gram-negative bacterial infections.

Other differentials to consider would include a nephrocolic fistula and iatrogenic injury to the urinary tract, although these were not likely in this case given the bilateral symmetrical emphysematous pyelonephritis, the patient’s medical history and the absence of recent surgery or instrumentation of the urinary tract.

## Treatment

This case demonstrated severe emphysematous pyelonephritis, with bloods suggesting evidence of a severe AKI and DIC secondary to sepsis. Although nephrectomy would be the first-line intervention in such a case, the patient was not clinically stable enough to undergo a major operation and was medically managed in the ICU, with commencement of broad-spectrum intravenous piperacillin/tazobactam antibiotics, antifungal therapies, and renal replacement therapy (RRT).

## Outcome and follow-up

In total, the patient was medically managed in the ICU for 41 days.

**Figure 3.**
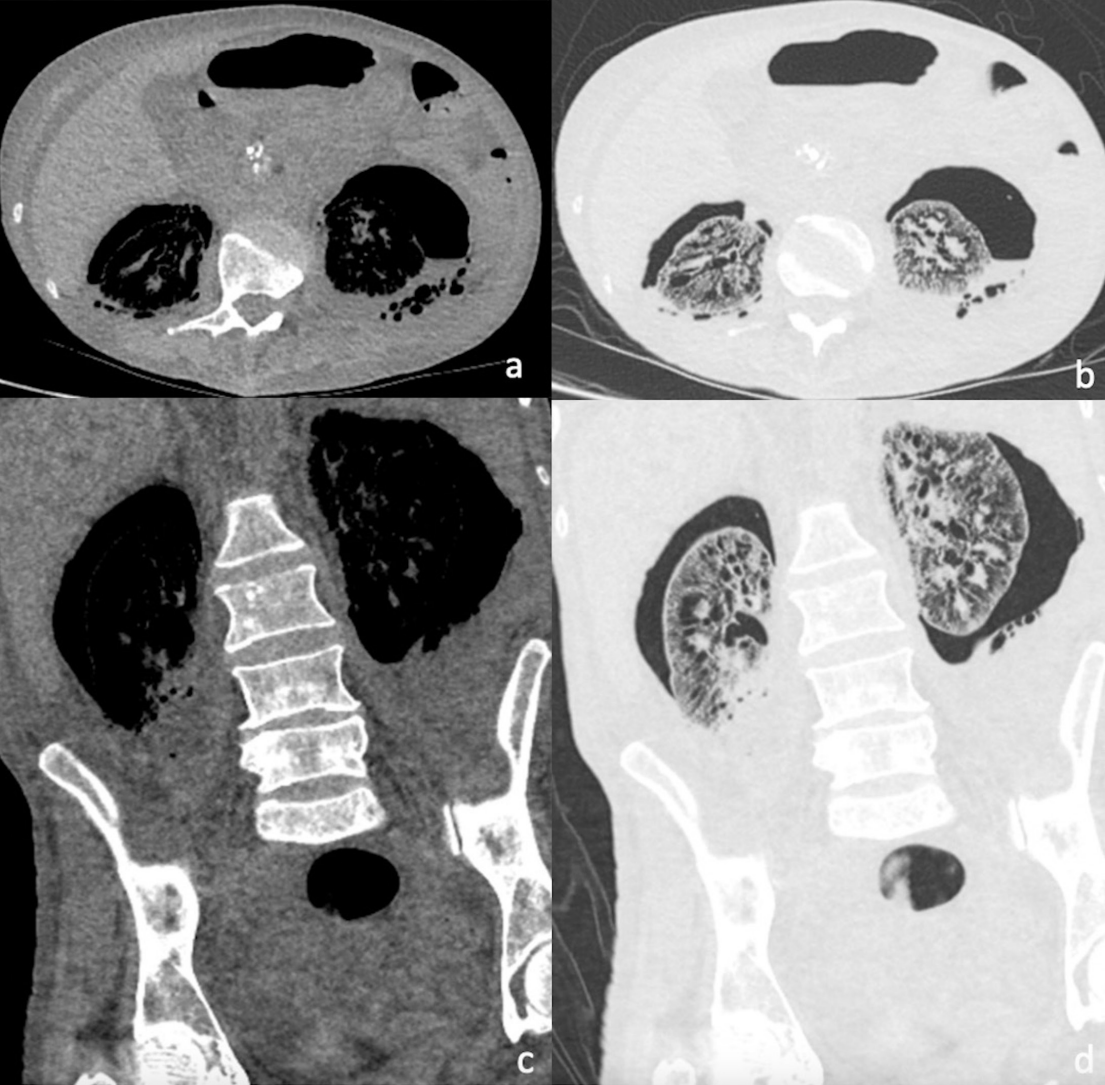
(a–d) Non-contrast CT abdomen and pelvis demonstrating similar appearances of the intrarenal gas collection to the previous scan but a marked increase in the perirenal gas collection, particularly around the left kidney. There is also increased gas in the collecting system on the right. (a) and (c) are set on soft tissue windows. (b) and (d) are on lung windows.

One week following admission, the patient clinically deteriorated. Progression of the renal infection was demonstrated through rising inflammatory markers and an increase in the volume of gas surrounding the kidneys seen on a subsequent CT scan (Figure 3a–d). Antibiotic therapy was escalated. Two weeks following admission, a repeat CT scan demonstrated evidence of an increase in the left-sided perirenal collection and potential splenic infarcts. The patient was therefore commenced on treatment–dose anticoagulation.

An attempt was made to wean the patient’s RRT; however, this led to an increased oxygen requirement of 4 L. This was attributed to pulmonary oedema secondary to reduced fluid removal. After 41 days in ICU, the patient was transferred to a specialist renal unit at a different hospital for initiation of life-long haemodialysis. The patient unfortunately passed away following this.

## Discussion

Emphysematous pyelonephritis is a rare but serious complication of diabetes mellitus, with a high mortality rate of around 25%.^4^ Less commonly, it has been seen as a complication of urinary tract obstruction or other causes of immunocompromise.^3^ The most common presenting symptoms are fever and flank pain, and other non-specific features of infection such as nausea, vomiting and hypotension.^5^ However, as demonstrated in our case, the diagnostic process may be complicated by other concurrent infections and sepsis that can present similarly. Therefore, new sepsis-associated AKI in diabetic patients should warrant a high index of suspicion for emphysematous pyelonephritis. This is particularly the case when there is a known infection with a gas-forming organism, the most common including *E. coli*, *Klebsiella pneumoniae* and *Proteus mirabilis*.^1,3^

There is no specific laboratory test to diagnose emphysematous pyelonephritis, and imaging is required. A CT scan has greater sensitivity than a plain film radiograph, and allows both delineation of the extent of the disease and identification of associated complications.^1^ Features on CT in severe cases such as ours are renal cortical destruction and replacement of renal tissue with gas, whilst in milder cases there may just be a few locules of gas in the renal parenchyma or perinephric space. The CT classification proposed by Huang and Tseng^1^ is widely used to describe the severity of emphysematous pyelonephritis based on the radiological presence of gas within different parts of the renal system. Whilst Class 1 describes gas limited to the collecting system, Class 2 is defined as the presence of gas in the renal parenchyma without extension to the extrarenal space. Class 3A and 3B describe extension of gas or abscess to the perinephric and pararenal spaces, respectively. Class 4 is the most severe stage and is reserved for patients with bilateral emphysematous pyelonephritis, as seen in our case, or a solitary kidney with emphysematous pyelonephritis. Other CT findings of complications include psoas abscess, emphysematous osteomyelitis and pneumomediastinum.^5^

Once diagnosed, prompt management is essential. These patients require supportive management in the ICU, including supplementary oxygen, I.V. hydration, glycaemic control, and correction of electrolyte imbalances. I.V. broad-spectrum antibiotics with Gram-negative cover are used for therapeutic management, and in severe cases in conjunction with surgical source control.^6^ Since the latter entails nephrectomy, consideration needs to be given that the surgery represents a major undertaking for the acutely ill patient. Patients such as ours with bilateral emphysematous pyelonephritis in the setting of multiple comorbidities, frailty and malnutrition are unlikely to be surgical candidates.^7^ Interventional radiology plays a key role as an adjunct to medical therapy for percutaneous drainage of infected collections.^8^

In cases such as ours, where there has been severe renal destruction during the acute illness phase, the patient is committed to life-long haemodialysis after recovery. A thorough exploration of social support structures, access to healthcare, and patient willingness are essential. Psychological and social worker support where indicated should be considered.^9^

In summary, severe emphysematous pyelonephritis can be a difficult diagnosis to make in the complex co-morbid patient, but one with severe life-threatening and life-altering prognosis. Early diagnosis and treatment are essential, as is involvement of a multidisciplinary team of, *e.g.* renal physicians, microbiologists, surgeons, social workers and psychologists.

## Learning points

- A high index of suspicion is required for emphysematous pyelonephritis in patients with sepsis and AKI in the setting of diabetes mellitus.
- Patients known to have poor glycaemic control and non-compliance to medical advice present unique challenges for the management of diabetes mellitus and are at particular risk of emphysematous pyelonephritis.
- In the absence of specific laboratory tests and symptoms, recognition of subdiaphragmatic retroperitoneal gas on a chest radiograph should prompt early acquisition of CT imaging when there is clinical concern.
- Once diagnosed, management decisions entail consideration of the patient’s social circumstances, support system, and baseline well-being. In severe cases, both bilateral nephrectomy and lifelong haemodialysis are major life-altering undertakings even if the patient recovers from the acute illness.
